# The roles of the ubiquitin-proteasome system in renal disease

**DOI:** 10.7150/ijms.107284

**Published:** 2025-03-10

**Authors:** Danqin Lu, Yingying Zhang, Ping Zhu, Jiao Wu, Cheng Yuan, Lihua Ni

**Affiliations:** 1 Department of Nephrology, Zhongnan Hospital of Wuhan University, Wuhan, Hubei, China.; 2 Department of Nephrology, Tongii Hospital of Tongji University, Shanghai, China.; 3 Division of Nephrology, The First College of Clinical Medical Science, Three Gorges University, Yichang, Hubei, China.; 4 Department of Nephrology, Affiliated Renhe Hospital of China Three Gorges University, Yichang, Hubei, China.; 5 Department of Oncology, Yichang Central People's Hospital and The First College of Clinical Medical Science, China Three Gorges University Yichang, Hubei, China.; 6 Tumor Prevention and Treatment Center of Three Gorges University and Cancer Research Institute of Three Gorges University Yichang, Hubei, China.

**Keywords:** Ubiquitin-proteasome system, deubiquitinases, autophagy, renal disease, diabetic kidney disease

## Abstract

The ubiquitin-proteasome system (UPS) is a major pathway of specific intracellular protein degradation through proteasome degradation of ubiquitin-labeled substrates. Numerous biological processes, including the cell cycle, transcription, translation, apoptosis, receptor activity, and intracellular signaling, are regulated by UPS. Alterations of the UPS, which render them more or less susceptible to degradation, are responsible for disorders of renal diseases. This review aims to summarize the mechanism of UPS in renal diseases. Besides, this review explores the relationship among UPS, autophagy, and deubiquitination in the development of renal disease. The specific molecular linkages among these systems and pathogenesis, on the other hand, are unknown and controversial. In addition, we briefly describe some anti-renal disease agents targeting UPS components. This review emphasizes UPS as a promising therapeutic modality for the treatment of kidney disease. Our work, though still basic and limited, could provide options to future potential therapeutic targets for renal diseases with a UPS underlying basis.

## Introduction

Proteins play a crucial role in living organisms' structural integrity and physiological functions. Within organisms, proteins undergo a continuous process of degradation into amino acids and synthesis of new proteins to sustain normal physiological processes. The degradation of proteins primarily occurs through the UPS and autophagosomal lysosomal system (ALS), with the UPS responsible for the degradation of approximately 80% of proteins, particularly those that are short-lived, regulatory, damaged, or misfolded^1^. Different proteins are degraded at different rates, ranging from a few minutes for metabolic enzymes, weeks for actin and myosin, to months for hemoglobin^2^. Organisms possess intricate regulatory mechanisms to ensure the selective degradation of proteins, thereby maintaining protein homeostasis. In recent years, more and more studies on UPS have been conducted, and we have recognized that UPS can play an important role in various renal diseases such as renal cell carcinoma^3^, acute kidney injury^4^, and renal fibrosis^5^ through a variety of mechanisms. The pathophysiological processes of renal diseases are regulated by the intricate relationship between UPS and ALS, the balance between ubiquitination and deubiquitination^6^. In this review, we summarize the recent studies on UPS in renal diseases, introduce the role and mechanism of UPS in renal diseases, and provide new strategies for the future treatment of renal diseases.

## Ubiquitin-proteasome system (UPS)

**Figure 1:**
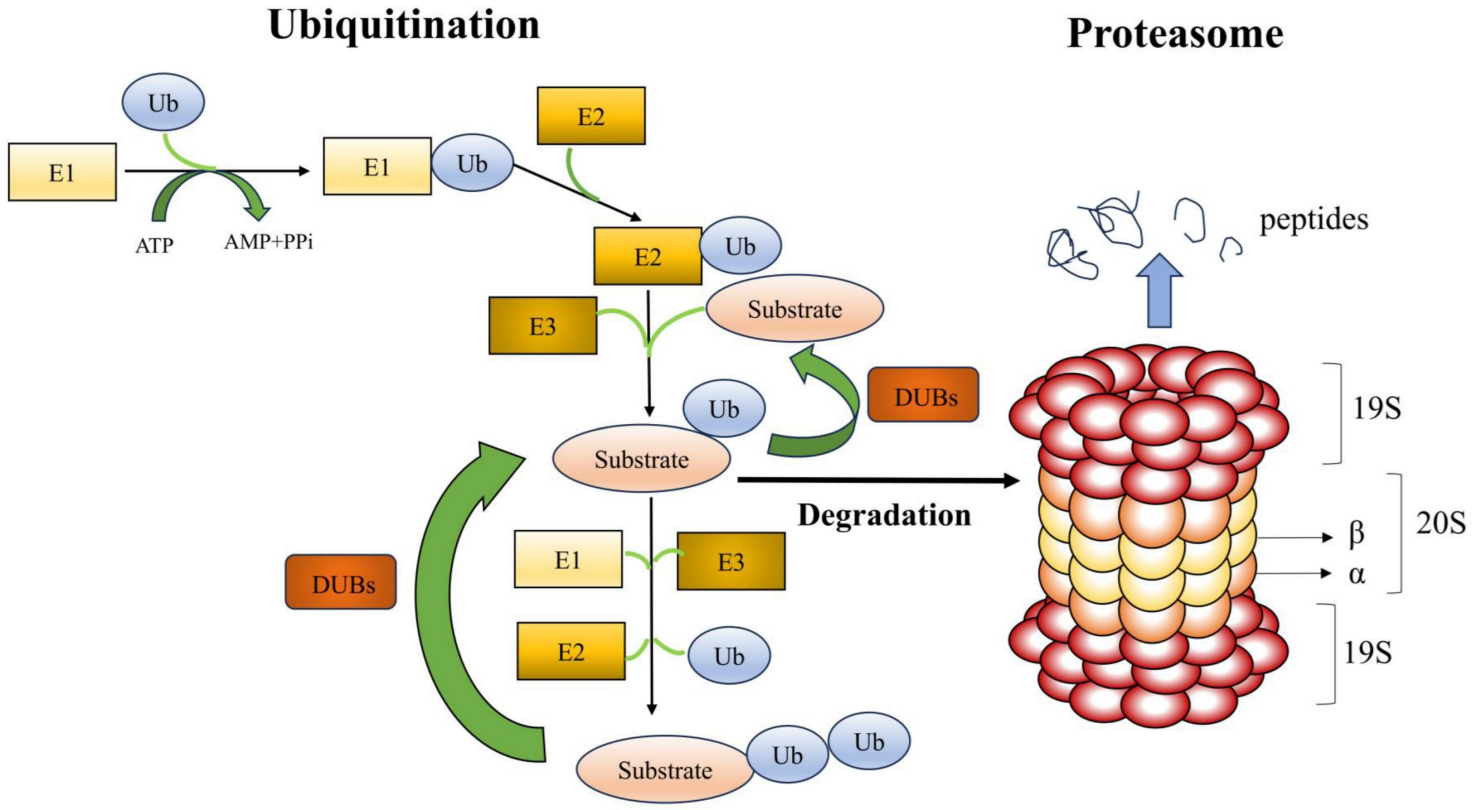
** Ubiquitin-proteasome system (UPS).** With three ubiquitinases, ubiquitin is bound to the lysine residues of substrate proteins and consumes ATP as energy. The two primary components of the proteasome are the 20S core particle (CP) and the 19S regulatory particle (RP). The RP is in charge of identifying, unfolding, and transferring polyubiquitylated substrates into the protein hydrolysis lumen inside the CP, while the CP is principally in charge of hydrolyzing proteins. Deubiquitinating enzymes identify and excise the ubiquitin chains attached to the substrate proteins.

UPS is made up of the ubiquitination and proteasome system (**Figure 1**). A substrate protein and ubiquitin are covalently linked during the enzymatic reaction process known as ubiquitination. A highly conserved 76 amino acid protein molecule called ubiquitin (Ub) attaches to substrate proteins to alert the proteasome to their breakdown. Three enzymes are involved in the ubiquitination process: E1 (ubiquitin-activating enzyme), E2 (ubiquitin-carrying or conjugating proteins), and E3 (ubiquitin-protein ligase). Ub is activated by E1, which hydrolyzes ATP and binds Ub to its cysteine residues, as the initial stage in the binding of Ub to substrate proteins. After that, the active ubiquitin is transported to the cysteine residue of E2. The E3 ligase acts as a docking platform to attach the Ub-containing E2 enzyme to a particular substrate, enabling the Ub to bind to the lysine residue of the substrate protein. All mammalian cells can activate ubiquitin thanks to a single E1 enzyme. It has been found that fifty E2 enzymes are involved in ubiquitin's coupling to substrate proteins. Over 1,000 types of E3 ligases have been identified, and this protein is primarily responsible for the specificity of ubiquitination^2^. E3 ligases identify particular substrate proteins and help Ub's binding to those substrate proteins. Based on structural similarities, the mode of ubiquitin transfer, and the functional class of substrate they recognize, E3 ligases are grouped into three primary families: the RING, HECT, and RBR families. While HECT and RBR E3 ligases first couple active ubiquitin to an E3 ligase before transferring it to the substrate, RING E3 ligases catalyze the direct transfer of ubiquitin from E2 to the substrate. There are three types of ubiquitination: mono-ubiquitination, in which one ubiquitin molecule binds to a substrate protein; multiple mono-ubiquitination, in which multiple lysine residues of a substrate protein are tagged by several mono-ubiquitin molecules at the same time; and polyubiquitination, in which the C-terminal glycine of a ubiquitin chain formed by several ubiquitin molecules binds specifically to a mono-ubiquitinated substrate^7^. The most common types of ubiquitination are mono-ubiquitination, K48-linked, and K63-linked polyubiquitination. Mono-ubiquitination is associated with protein recognition or regulation of aliasing, whereas K48- and K63-linked polyubiquitination may be associated with protein degradation and signal transduction, respectively^8^. Both K48- and K63-linked polyubiquitination can play a role in kidney disease. It has been shown that E3 ligase retinoblastoma binding protein 6 (RBBP6) promotes k48-linked ubiquitination of estrogen-related receptor α (ERRα), exacerbates mitochondrial damage in proximal renal tubular cells, promoting the development of diabetic kidney disease^9^. The rat Thy-1 nephritis model is an experimental model of human mesangioproliferative glomerulonephritis (MsPGN). In this model, the E3 ligase tumor necrosis factor receptor-associated factor 6 (TRAF6) promotes k63-linked ubiquitination of krϋppel-like transcription factor 5 (KLF5) and facilitates the interaction of KLF5 with downstream molecules, which provides a new possibility for a therapeutic approach to MsPGN^10^. In conclusion, prior research has demonstrated that k48- and k63-linked polyubiquitination may play a role in the development of many renal disorders; however, the precise mechanisms remain to be further explored.

The proteasome recognizes and degrades ubiquitinated proteins. The core particle (CP; also called the 20S CP) and the regulatory particle (RP; also called the 19S particle or PA700) make up the proteasome^11^. Protein hydrolysis is carried out by CP, while RP is in charge of identifying, unfolding, and transferring ubiquitinated substrates into the CP's protein hydrolysis cavity. CP is a cylinder-shaped structure with two outer rings (α) and two inner rings (β), which has four heptameric rings with seven α or seven β subunits^12^. The amino-terminal ends of the seven α-subunits function as a portal for the substrate to enter the protein hydrolysis chamber, which is formed by β-rings. β1, β2, and β5 exhibit proteolytic hydrolysis activities; β1 exhibits aspartate cysteine-like hydrolysis, β2 trypsin-like hydrolysis, and β5 chymotrypsin-like hydrolysis^13^. RP is made up of a lid and a bottom. The non-ATPase is on the lid, while the ATPase is at the bottom. The ATPase is responsible for mediating the binding, unfolding, and translocation of the substrate and the opening of the CP gate. To help with the initial binding of ubiquitinated substrates, the non-ATPase can function as a ubiquitin receptor. A CP with one or two RPs can be found in the proteasome, resulting in the asymmetric 26S proteasome or 30S proteasome, respectively^14^. The most common type of proteasome isoform in mammals is 26S constitutive proteasomes. The β1, β2, and β5 subunits in the CP can be substituted with β1i, β2i, β5i, β5t, and α4s; β1i, β2i, and β5i subunits are found in immunoproteasomes; β1i, β2i, and β5t subunits are found in thymoproteasomes; and β4s subunit is present in the testis proteasome^15^. When proteins tagged with ubiquitin bind to the proteasome, the ubiquitin tag is removed and the proteins are broken down into smaller pieces.

## Roles of the ubiquitin-proteasome system in renal diseases

### Acute kidney injury (AKI)

Acute kidney injury (AKI) is a clinical syndrome that can arise from a variety of factors that produce a quick loss in renal function over a brief period. Effective and precise treatments are still lacking, because the pathophysiology of AKI is intricate. Ischemia-reperfusion injury (IRI), sepsis, and cisplatin are common causes of AKI that may lead to irreversible damage to renal tissue.

IRI, sepsis, and cisplatin are also commonly used methods to construct AKI models in current research. By creating an *in vivo* model, these three approaches can as closely mimic the pathological process of human AKI as possible; nonetheless, there are a few differences across the models. The IRI model is the most widely used prerenal AKI model, and the severity of AKI is influenced by the length of ischemia and reperfusion. Both sepsis and cisplatin-induced AKI are renal AKI. Sepsis AKI is mainly characterized by cecal ligation and puncture (CLP) and lipopolysaccharide (LPS). Although the CLP-induced AKI model is similar to the clinical pathogenesis, the challenge of standardizing the process could result in variations in AKI models between studies, as well as a higher risk of infection and less repeatability in the CLP model. The LPS model is simpler, but different bacterial sources of LPS as well as the dosage of LPS and the way of administering LPS may affect the success of modeling and the severity of AKI. The dose and method of cisplatin administration are the primary factors that determine the effectiveness of the cisplatin-induced AKI model, but the sensitivity to cisplatin may vary among animal strains.

#### NF-κB

Through activation of NF-κB, UPS has been reported to stimulate the formation of endothelin 1 (ET-1)^16^. ET-1 is a potent local vasoconstrictor and may be a significant cause of IRI. The two subunits of NF-κB are p50 and p 65. When NF-κB is at rest, it is inactive and is stored in the cytoplasm with IκB, an NF-κB inhibitor. When IRI occurs, NF-κB is activated in the cytoplasm. The UPS degrades the p105 precursor protein of NF-κB to mature subunits. And the two serine residues of IκB (α and β) are ubiquitylated and degraded resulting in the dissociation of the NF-κB active units, p50/p65 dimer, from IκB α and β, and exposing the active site of NF-κB. After activation, NF-κB rapidly translocates to the nucleus and binds specifically to target genes, thereby initiating the transcription of Endothelin-1 (ET-1) and inflammatory cytokines, such as tumor necrosis factor (TNF), Interleukin-1β (IL-1β), and Interleukin-6 (IL-6)^17^.

The deubiquitinating enzymes breast cancer susceptibility protein 1 (BRCA1)-associated protein 1 (BAP1) and USP9x are also involved in sepsis-induced AKI through the NF-κB signaling pathway. In a model of AKI generated by cecal ligation and puncture (CLP), the expression of BAP1 was much lower. The deubiquitination modification of BRCA1 is reduced by BAP1 in AKI, which significantly decreases the stability of BRCA1. Reduced BRCA1 activates the NF-κB signaling pathway, which contributes to tissue damage and the inflammatory response in AKI. In mice, up-regulation of BAP1 delays AKI and preserves renal tissues^18^. In a sepsis-induced AKI model, USP9x inhibits the ubiquitinated degradation of toll-like receptor 4 (TLR4), which is involved in inflammatory responses and apoptosis by activating the NF-κB signaling pathway through interaction with lipopolysaccharide (LPS). Inhibition of USP9x expression can delay the progression of AKI and protect renal function^19^.

#### Ferroptosis

UPS can also be involved in the development of AKI by regulating ferroptosis. In the ischemia-reperfusion (I/R)-AKI model, the E3 ligase Trim21 is highly expressed, which modulates AKI by ubiquitinating degradation of glutathione peroxidase 4 (GPX4). By transforming lipid peroxides into inert lipid alcohols, GPX4 inhibits ferroptosis. Trim21 participates in AKI through downregulating GPX4, which causes oxidative stress and promotes ferroptosis in renal tubular cells^20^. In the I/R rat model, expression of the deubiquitinating enzyme USP7 is increased. USP7 promotes E3 Ub-protein ligase Trim27-mediated ubiquitination degradation of TANK-binding kinase 1(TBK1). USP7 deubiquitinates DNA methyltransferase (DNMT1), resulting in increased stability of DNMT1. By recruiting at the FMRP translational regulator 1 (FMR1) promoter, DNMT1 suppresses the expression of FMR1 by promoting promoter methylation. TBK1 can also affect the expression of FMR1. Reduced expression of FMR1 regulates AKI and promotes ferroptosis^21^.

Ferroptosis has also been shown to play an important role in other AKI models. In a CLP-induced mouse model of AKI, the E3 ligase MDM2 increased the expression of LMNB1 by promoting the ubiquitinated degradation of p53.LMNB1 may promote mitochondrial damage and ferroptosis in renal tubular epithelial cells and be involved in the development of AKI^22^. CD36 expression was markedly increased in the cisplatin-induced model of AKI. CD36 contributed to the development of AKI by ubiquitinated degradation of the ferroptosis suppressor protein 1 (FSP1) and promoting ferroptosis in renal tubular cells^23^.

#### Oxidative stress

Oxidative stress is an important feature of AKI, leading to mitochondrial damage and promoting inflammatory responses. The deubiquitinating enzymes USP10 and USP36 and the E3 ligase circRNA itchy E3 ubiquitin-protein ligase (circ-ITCH) are involved in the development of AKI through oxidative stress. The nuclear factor E2-related factor2/antioxidant response elements (Nrf2/ARE) signaling pathway is an important signaling pathway to resist oxidative damage. In a mouse model of sepsis-induced AKI, the expression of the deubiquitinating enzyme USP10 was decreased. By deubiquitinating Sirt6, USP10 promotes the Nrf2/ARE signaling pathway, preserves renal function, and attenuates tubular epithelial cell injury and oxidative stress in mice^24^. The expression of USP36 is reduced in AKI patients and mouse models. Superoxide dismutase 2 (SOD2) is an antioxidant enzyme that ameliorates oxidative stress by converting superoxide to hydrogen peroxide. C-Myc regulates cell proliferation and differentiation after AKI and promotes renal repair after damage. USP36 was found to protect renal function by deubiquitinating SOD2 and c-Myc, attenuating oxidative damage, and promoting cell proliferation^25^. In a mouse model of sepsis, the expression of circ-ITCH was decreased. Circ-ITCH could protect renal function by targeting miR-214-3p, promoting the expression of ABCA1, and inhibiting oxidative stress and mitochondrial damage^26^.

#### Lipid homeostasis

In an acute kidney injury model, the expression of α Klotho on renal tubular epithelial cells is decreased. By encouraging the interaction of the E3 ligase peroxin2 (PEX2) with the E2 ubiquitin-conjugating enzyme D2 (UBC4), α Klotho enhances the ubiquitinated degradation of adipose triglyceride lipase (ATGL), a crucial enzyme in lipolysis and lipoautophagy. The decreased expression of ATGL inhibits fatty acid mobilization of intracellular lipid droplets and promotes lipid droplet's aggregation, leading to renal tubular epithelial cell injury and renal fibrosis, and ultimately promotes the transformation of AKI to CKD^27^.

#### KLF4/ITCH/LATS1/YAP

Studies have shown that yes-associated protein (YAP) promotes renal tubular epithelial cell repair and protects renal function in the acute phase of AKI. However, in the post-acute phase, YAP can promote the conversion of IR-induced AKI to CKD. Increased expression of the transcription factor krϋppel-like transcription factor4 (KLF4) promotes the expression of the E3 enzyme itchy E3 ubiquitin-protein ligase (ITCH).ITCH activates YAP by promoting the ubiquitination of the phosphatase large tumor suppressor 1 (LATS1).YAP can promote renal fibrosis after AKI by promoting the expression of transforming growth factor-β (TGF-β) and connective tissue growth factor (CTGF)^28^.

Certain proteasome inhibitors have been studied in AKI based on the function of UPS in AKI. The proteasome inhibitor lactacystin was applied to a rat model of IRI, which resulted in a decrease in ET-1 levels and a delay in the decline of renal function^29^. The proteasome inhibitor Z-Ile-Glu (Ot-Bu) Ala-Leucinal (PSI) inhibited venous thrombosis, platelet adhesion aggregation, and ischemic renal insufficiency in rats in a model of renal hypertension^30^. In conclusion, UPS has an important role in the development of AKI, mostly by NF-κB, ferroptosis, oxidative stress, lipid homeostasis, and KLF4/ITCH/LATS1/YAP pathway. However, these studies are currently preclinical trials, and large-scale clinical studies are still needed to further explore the critical role of targeted USP in AKI treatment.

### Diabetic kidney disease (DKD)

As one of the most common microvascular complications of diabetes, diabetic kidney disease (DKD) is a condition of renal structural and functional damage caused by persistent hyperglycemia. It has an increased risk of renal failure. Impairment of the structure and function of podocytes, renal tubular epithelial cells, and glomerular mesangial cells is central to the pathogenesis of DKD. In podocytes cultivated in high sugar conditions, there was an increase in ubiquitinated proteins and a suppression of proteasome which induced apoptosis^31^. Additionally, high sugar directly glycosylates the podocyte's proteasome, which lowers proteasome activity^32^. This implies that the physiological function of the kidney depends on the accurate regulation of UPS and that abnormalities in UPS activity and composition will result in damage to the kidney. Research has demonstrated that elevated immune proteasome expression in podocytes in DKD may cause autoimmune disorders and cellular inflammation.

#### Podocyte

Podocyte injury is an important cause of kidney damage and proteinuria in patients with DKD. Through the regulation of glucose-6-phosphate dehydrogenase (G6PD), carnitine palmitoyl transferase 1a (CPT1A), acyl-coenzyme A oxidase 1(ACOX1), NF-κB, and cell cycle protein activities, E3 Ub-protein ligase affects podocytes' function and plays a significant role in the development of DKD. In high glucose conditions, the expression of E3 ligase VHL was increased in podocytes. VHL ubiquitinates glucose-6-phosphate dehydrogenase (G6PD) at K366 and K403, resulting in increased degradation of G6PD by the proteasome. The decreased expression of G6PD causes reactive oxygen species accumulation and redox imbalance, which ultimately leads to increased apoptosis in podocytes^33^. The E3 ubiquitin ligase Trim63 (also known as MuRF1) can regulate the activity of key enzymes involved in fatty acid oxidation (FAO), such as CPT1A and ACOX1, by affecting the activity of peroxisome proliferator-activated receptor α (PPARα). Trim63 facilitates the ubiquitinated degradation of PPAR α in the diabetic kidney disease model caused by adriamycin (ADR). Decreased PPAR α aggravates proteinuria and renal damage by causing mitochondrial dysfunction and fatty acid buildup, which in turn increases podocyte death^34^. The E3-ubiquitin ligase NEDD4L stimulates the IκB kinase complex (IKK) to be ubiquitinated and degraded. It facilitates NF-κB entry into the nucleus and transcription, which activates the IKK/NF-κB signaling pathway, intensifying podocyte damage and the inflammatory response, and contributing to the development of DKD^35^. Trim29 interacts with IκBα (NF-κB inhibitory protein), mediating its ubiquitination-dependent degradation, which results in NF-κB activation. This activation then triggers the transcription of the nucleotide-binding oligomerization domain, leucine-rich repeat, and pyrin domain-containing protein 3 (NLRP3) gene, causing podocyte pyroptosis and contributing to the development of DKD^36^. The cell cycle proteins S-phase kinase-associated protein-2 (Skp2) and Cyclin B1 are ubiquitinated and degraded by the E3 ligase APC/C. In the kidneys of diabetic mice and patients, the expression of mitotic arrest deficiency 2B (MAD2B) is upregulated. MAD2B inhibits the activity of E3 ligase APC/C by suppressing Cdh1 expression. Decreased activity of APC/C leads to increased expression of Cyclin B1 and Skp2, which further mediates podocyte injury^37^.

#### Renal tubular epithelial cell (RTEC)

The injury of renal tubular epithelial cells (RTECs) and epithelial-mesenchymal transition (EMT) are the main causes of renal function decline in DKD patients. It was discovered that UPS controls the function of RTECs via the transcription factors sterol regulatory element-binding protein 1 (SreBP-1), GATA4/GSA1 route, STAT1/TGF-β1 pathway, and PINK1/Parkin pathway. Studies have shown that tumor necrosis factor alpha-induced protein 8-like 1 (TNFAIP8L1/TIPE1) could regulate PINK1/Parkin-mediated mitochondrial autophagy to protect renal function in DKD. Both STZ-induced diabetic mice and the RTECs of diabetic patients express higher levels of TIPE1, and renal damage and renal mesenchymal fibrosis are lessened by tipel-specific silencing^38^. Prohibitin2 (PHB2) is a mitochondrial autophagy receptor that binds to LC3 in mammalian cells. PHB2 is a critical regulator of Parkin-induced mitochondrial autophagy and a mitochondrial stabilizer of PTEN-induced kinase1 (PINK1). Increased expression of TIPE1 in DKD leads to the ubiquitinated degradation of PHB2, which disturbs mitochondrial homeostasis, inhibits PINK1/Parkin-mediated mitochondrial autophagy, and encourages EMT and kidney damage. In RTECs grown in high glucose, the expression of the E3 ligase Rffl was decreased, which activated the STAT1/TGF-β1 signaling pathway. TGF-β1 also positively regulated the extracellular matrix (ECM) and led to significant up-regulation of the ECM marker proteins alpha smooth muscle actin (αSMA) and zinc finger E-box-binding homeobox protein1(ZEB1) and down-regulation of E-cadherin, which in turn encouraged renal mesangial fibrosis and glomerulosclerosis^39^. E3-ubiquitin ligase Parkin reduces the course of DKD by promoting GATA4 ubiquitination, downregulating the GATA4/GSA1 signaling pathway, and preventing inflammation, fibrosis, and premature senescence of RTECs^40^. The F-box and WD repeat domain -containing 7 (FBXW7) is the largest E3 ubiquitin ligase family Skp1-Cullin1-F-box (SCF) complex, which affects a variety of biological processes including cell proliferation, apoptosis, and differentiation. Under high glucose stimulation, the decreased expression of FBXW7 in RTECs may mediate the increased expression of SreBP-1, which affects lipid metabolism. SreBP-1 is an important transcription factor in lipid metabolism. Up-regulating SreBP-1 expression raises the activities of fatty acid synthase and acetyl coenzyme A carboxylase, which stimulates the production of fatty acids and cholesterol and causes abnormal lipid buildup in renal tubular cells^41^.

#### Glomerular mesangial cells (GMCs)

The abnormal function of glomerular mesangial cells (GMCs) plays a significant role in the etiology and course of DKD. Previous studies have demonstrated that UPS affects the function of GMCs through AGES/RAGE, NF-κB, Trim13/CHOP, Smurf1/TGR5, and Nrf2/ARE pathways. Advanced glycation-end products (AGEs) are produced by persistent glucose metabolism disorders in diabetic states. When AGEs bind to receptors known as RAGEs, they activate inflammatory signaling pathways like NF-κB and mitogen-activated protein kinase (MAPK) and encourage the expression of fibrotic markers like TGF-β1 and fibronectin (FN) in GMCs. Further studies revealed that USP22 regulated the ubiquitination and proteasomal degradation of silent information regulator 2-related protein 1 (Sirt1) in GMCs, modulated the AGEs/RAGE pathway, and stimulated the expression of FN and TGF-β1, which accelerates the fibrosis process in diabetic kidneys^42^. USP25 also protects renal function by modulating the AGEs/RAGE pathway. In kidneys with diabetic renal disease, USP25, a deubiquitinating enzyme, is mostly expressed in GMCs and macrophages. USP25 blocks the process of TRAF6 recruitment and activation of TAK1 by inhibiting the K63-polyubiquitination process of TRAF6. TAKI functions as an upstream signaling molecule for both NF-κB and MAPK. The AGEs-RAGE system-induced MAPK and NF-κB signaling pathways are compromised when TAK1 activation is blocked^43^. By preventing the polyubiquitination of phosphorylated IκBα, the deubiquitinating enzyme CYLD protects GMCs by suppressing the activation of NF-κB signaling in a high-glucose environment^44^. By interacting with C/EBP homologous protein (CHOP), the E3 ligase Trim13 promotes collagen synthesis and ECM accumulation in GMCs. ECM thickens the glomerular basement membrane and promotes renal fibrosis. In GMCs, CHOP is linked to the expression of collagen-related factors such as Col1a2, TGF-β1, and Col1a4, which promote the synthesis of collagen in DKD. Increased levels of DNA methylation of the Trim13 gene promoter in DKD result in lower production of Trim13, decreased proteasomal degradation and ubiquitination of CHOP, and increased collagen synthesis^45^. In GMCs, it was shown that the E3 ubiquitin ligase Smurf1 interacted with TGR5 to promote the ubiquitination and proteasomal degradation of TGR5. Conversely, in diabetic mice, Smurf1 deficiency reduced renal damage and increased TGR5 levels in the kidney^46^. The Nrf2/ARE signaling pathway is an important endogenous oxidative stress pathway in the body and inhibits renal fibrosis in DKD. The Nrf2/ARE signaling pathway is inhibited by elevated expression of Smurf1 in DKD, which raises ROS levels in GMCs and promotes renal fibrosis^47^.

**Figure 2:**
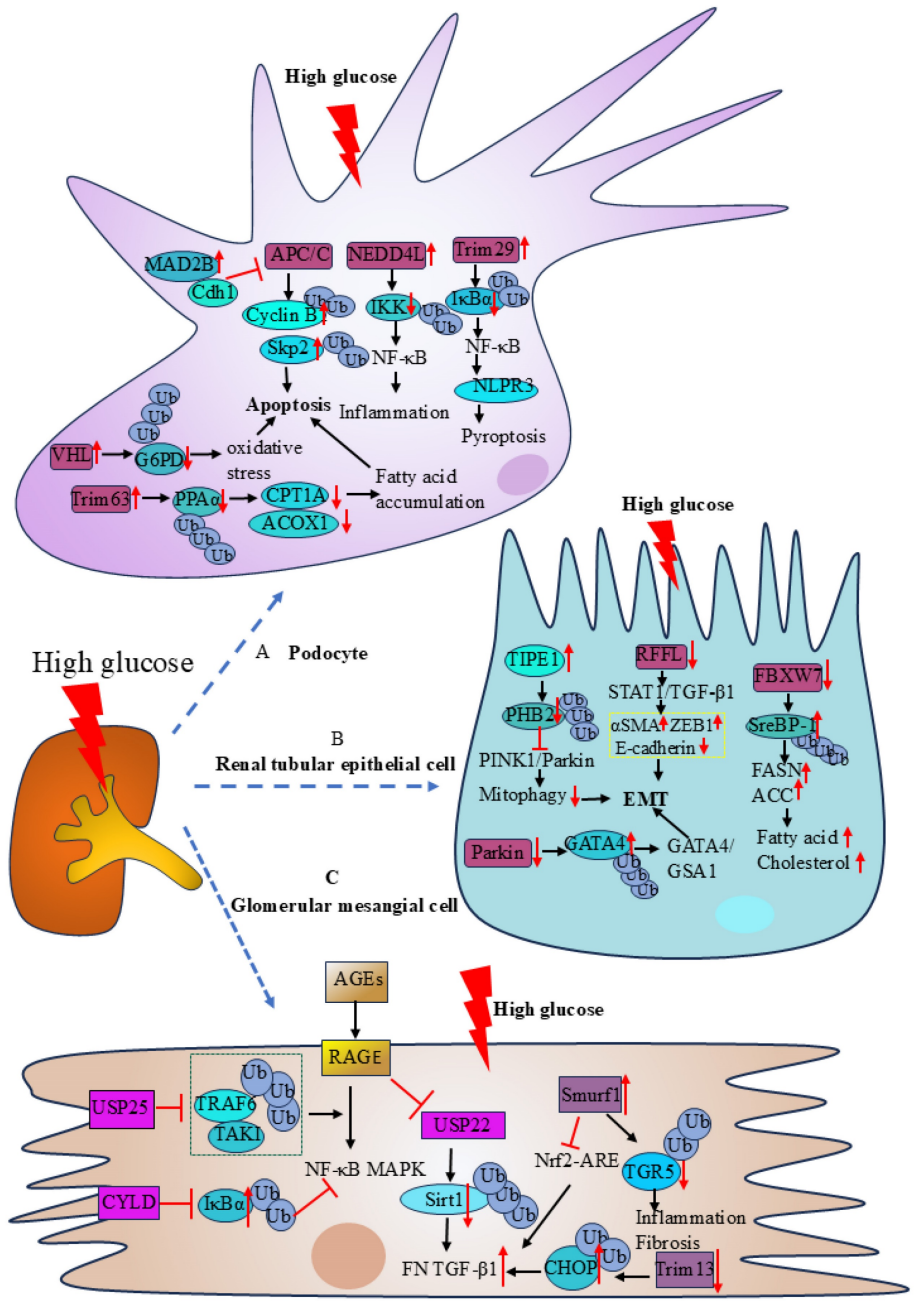
** The role of podocytes, renal tubular epithelial cells, and glomerular mesangial cells in DKD.** A.| High hyperglycemia stimulated the expression of Trim63 and VHL, which enhanced substrate ubiquitination and promoted podocyte apoptosis. The E3 ligase APC/C is inhibited by an increase in MAD2B level, which results in podocyte apoptosis. Both Trim29 and NEDD4L enhanced substrate ubiquitination degradation triggered the NF-κB signaling pathway and resulted in podocyte pyroptosis and inflammation, respectively. B.| Under the influence of elevated glucose, RTECs undergo EMT induced by TIPE1 and the E3 ligases Parkin and RFFL. Reduced FBXW7 expression raises substrate content, which in turn raises the activity of important lipid metabolism enzymes and causes abnormal lipid buildup in RTECs. C.| Deubiquitinase USP25 and CYLD can inhibit the NF-κB and MAPK signaling pathways caused by AGEs-RAGE, and inhibit the inflammatory response of GMCs. USP22, Trim13, and Smurf1 can cause extracellular matrix aggregation and promote renal fibrosis.

In conclusion, E3 Ub-protein ligase is involved in processes such as inflammatory response and injury in podocytes, renal tubular epithelial cells, and glomerular mesangial cells by affecting the activity of multiple signaling pathways (**Table 1**) (**Figure 2**). It is anticipated that additional studies to clarify the precise role of E3 ligases in DKD will result in the development of novel medications that specifically target E3 ligases for the treatment of DKD.

### Renal fibrosis

Renal fibrosis is the final result of several chronic kidney disorders, characterized by renal damage, fibrosis, and sclerosis brought on by a range of factors. Although its pathogenesis is unclear, available studies suggest that UPS is involved in renal fibrosis through inflammatory responses, oxidative stress, and the cell cycle.

#### TGF-β

TGF-β plays a key role in renal fibrosis, leading to tubulointerstitial fibrosis and glomerulosclerosis by promoting the production of inflammatory factors and fibroblast proliferation. TGF-β receptors, also known as TGF-βR1/TGF-βR2, are transmembrane receptors for serine/threonine kinases. TGF-βR1 and TGF-βR2 work together to create a receptor complex after binding to TGF-β, and TGF-βR2 phosphorylates TGF-βR1. After TGF-βR1 is phosphorylated, Smad2 and Smad3 are phosphorylated downstream. These phosphorylated Smad2 and Smad3 then interact with their co-chaperone Smad4 to form a complex that reaches the nucleus and stimulates the transcription of pro-fibrotic genes^48^. A transcriptionally inactive complex can be formed by Smad2 and Smad3 binding to transcriptional co-repressor proteins like SKI and SNON (also referred to as SKIL). Smad7 is an inhibitory Smad that binds to the TGF-β receptor to block Smad2 and Smad3 from binding, therefore inhibiting the TGF-β/Smad signaling cascade. UPS participates in both pro- and anti-fibrotic processes through the degradation of several TGF-β/Smad signaling pathway constituents. E3 ligases (Smurf1 and Smurf2) mostly function on Smad. When Smurf2 interacts with SNON, SKI, and Smad7, it causes renal fibrosis, but Smad2, Smad3, and Smad4 prevent renal fibrosis when they are ubiquitinated^49^.

#### Wnt / β-catenin

The Wnt/β-catenin signaling pathway in humans is rendered inactive by the β-catenin degradation complex, which phosphorylates β-catenin proteins and facilitates their ubiquitinated degradation. Renal disease and kidney injury trigger the activation of the Wnt/β-catenin signaling pathway. Dishevelled-2 (Dvl2) breaks apart the β-catenin degradation complex by binding to the Frizzled receptor and lipoprotein receptor-related protein-5/6 (LRP-5/6). Since β-catenin is not degraded by UPS, it may enter the nucleus and help the fibrogenic genes' transcription, which leads to the development of renal fibrosis^3^. The activity of this pathway is influenced by E3 Ub-protein ligase. In the IRI model, elevated expression of the E3 Ub-protein ligase HMG-CoA reductase degradation protein 1 (HRD1) increases ubiquitinated degradation of the β-catenin degradation complex, stabilizes β-catenin proteins, and thereby enhances Wnt / β-catenin signaling^50^. In contrast, the E3 Ub-protein ligase NEDD4L degrades Dvl-2, thereby inhibiting Wnt / β-catenin signaling and delaying renal fibrosis^51^.

#### PINK1 / Parkin

The PINK1 / Parkin signaling pathway can affect renal fibrosis by influencing mitophagy. PINK1 enters mitochondria and is quickly broken down. When mitochondria are injured, PINK1 builds up at the outer membrane of the damaged mitochondria, where it encourages the activation of Parkin, an E3 Ub-protein ligase, and translocation into damaged mitochondria. Parkin facilitates the process of ubiquitination of proteins in the outer membrane of damaged mitochondria, which triggers mitophagy through the autophagy-lysosome pathway. Chronic renal fibrosis advances more quickly in the DKD when PINK1/Parkin-mediated mitophagy is inhibited^52^. In cases of renal damage, suppression of the PINK1/Parkin signaling pathway causes a reduction in macrophage mitophagy. It encourages the differentiation of macrophages into pro-fibrotic M2 phenotypes and an increase in extracellular matrix synthesis, which exacerbates renal fibrosis.

#### Epidermal growth factor receptor (EGFR)

Epidermal growth factor receptor (EGFR) induces fibroblast proliferation and migration and participates in the renal fibrosis process through downstream signaling pathways such as TGF - β1 / Smad3, STAT3, and ERK1 / 2^53^. Patients with renal fibrosis exhibit increased expression of the deubiquitinating enzyme USP11, which positively correlates with the severity of renal fibrosis. USP11 stabilizes EGFR by inhibiting the ubiquitination degradation pathway. In the USP11 knockout mouse model of renal fibrosis, the degradation of EGFR was increased, which inhibited downstream signaling pathways and delayed the renal fibrosis and epithelial-mesenchymal transition^54^. In contrast, in the unilateral ureteral obstruction (UUO) animal model and clinical samples, the expression of the E3 ligase HUWEI was decreased and that of EGFR was increased. By promoting ubiquitinating degradation of the EGFR, HUWEI inhibits renal tubulointerstitial fibrosis and protects renal function^55^.

#### Checkpoint kinase 1 (CHK1)

RTECs that are blocked in the G2/M cell cycle are strongly linked to renal fibrosis. These cells can exacerbate renal fibrosis by producing pro-fibrotic factors. Checkpoint kinase 1 (CHK1) mediates cellular G2/M cycle block. The deubiquitinating enzyme USP7 promotes renal fibrosis by deubiquitinating CHK1^56^.

In conclusion, UPS is mainly involved in renal fibrosis through TGF-β, Wnt / β - catenin, and PINK1 / Parkin pathway and factors such as EGFR and CHK1. However, there are still many details to be explored about the specific mechanism of E3 ligases involved in renal fibrosis. Future therapeutic options can be explored on how to specifically target E3 ligases to delay renal fibrosis under the premise of maximum non-interference with normal cellular function.

### Renal carcinoma

In recent years, the incidence of renal cell carcinoma (RCC) has been gradually increasing and is second only to bladder cancer among urologic tumors. Renal cell carcinoma mainly consists of three subtypes: clear cell RCC (ccRCC), papillary type RCC (pRCC), and chromophobe RCC (chRCC), of which ccRCC accounts for more than 70% of the cases^57^. Existing studies have shown that UPS may be involved in the development of renal cancer. In renal cancer cells, increased expression of the E3 ligase UBE3C increased the expression level of Phosphatidylethanolamine Binding Protein 1 (PEBP1). And knockdown of UBE3C significantly suppressed the level of ubiquitination, suggesting that UBE3C may regulate the expression of PEBP1 through the UPS. The low expression of PEBP1 can affect ccRCC progression by affecting tumor angiogenesis by activating the extracellular regulated protein kinase (ERK) signaling pathway^58^. In renal cancer cells, the expression of the E3 ligase Trim65 is elevated, and overexpression of Trim65 promotes the growth of renal cancer cells. Trim65 promotes cell proliferation by promoting ubiquitination of the tumor suppressor gene BTG3, which promotes the expression of CyclinD1, a positive regulator of the cell cycle^59^. These studies suggest that E3 ligases regulate the development of RCC through multiple pathways, and further study of the mechanisms involved could help identify novel therapeutic options for RCC in the future.

Von Hippel-Lindau disease is a hereditary tumor syndrome and about 30%-70% of patients with VHL develop RCC, and VHL-related renal cell carcinoma is almost ccRCC. The tumor suppressor gene VHL is involved in the development of renal tumors, and early VHL tumor suppressor gene inactivation is present in over 60% of sporadic renal cell carcinomas. Together with elongin BC, CUL2, and RBX1, the tumor suppressor protein pVHL forms a functional E3 ligase complex. By binding to HIF-1α, pVHL facilitates its degradation through the UPS. The binding of VHL to elongin C is blocked in VHL gene-deficient clear cell renal cell carcinoma (ccRCC), which results in reduced ubiquitination and proteasomal degradation of HIF1-α. This leads to increased expression of the HIF-1α subunit HIF-1α3 and up-regulation of VEGF mRNA expression. These changes, along with its protein expression, significantly correlate with an increase in microvessel density, indicating that aberrant ubiquitination of HIF-1α may be linked to invasion and metastasis of VHL-deficient tumors^60^. Renal cell carcinoma cell lines lacking pVHL were no longer able to grow into tumors in mice when pVHL was reintroduced in these cell lines^61^. Additional studies have demonstrated that VHL mutations can act in a HIF-1α-independent way. Targets for clear cell renal cell carcinoma include the NF-κB agonists IκB kinase b and caspase recruitment domain protein 9 (CARD9), MAPK1, fibronectin, collagen IV, RpB1 (the large subunit of RNA polymerase II), human antigen R (HuR), and Akt (also known as protein kinase B)^62^. In kidney tumor cells 786-0 cells (cells with a deletion of one base pair on exon 1 of the VHL gene), the proteasome inhibitor Z-Leu-Leu-Leu-CHO (MG-132) promotes apoptosis in tumor cells. Proteasome inhibitor PS-341 inhibits NF-κB activity and promotes tumor cell apoptosis in renal cell carcinoma cell lines^63^. Due to the low incidence rate of Von Hippel-Lindau syndrome in the population, clinical studies on VHL have been limited to animal studies and retrospective case-control studies, and cohort studies are still needed to further validate the mechanisms involved.

### Liddle syndrome

Liddle syndrome is an autosomal dominant disorder, and its pathogenesis is unclear. Recent studies have found that UPS plays an important role in Liddle syndrome. The renal distal tubule and collecting duct epithelial cells' luminal membrane contains the sodium channel (EnaC), which is made up of α, β, and γ subunits. The WW structural domain on Nedd4-2, a member of the HECT E3 ligase family, recognizes the PY motif contained in the α, β, and γ subunits. It was shown that targeting Nedd4-2 would promote cellular EnaC expression and exacerbate Liddle syndrome^64^. Mutations in amino acids on the β and γ subunits lead to a decrease in the degradation of EnaC by UPS, which increases the amount of EnaC on epithelial cells, promotes water and sodium reabsorption, and facilitates the development of Liddle syndrome. Studies suggest that aldosterone can also affect EnaC expression via Nedd4-2. Serum/glucocorticoid-regulated kinase 1 (SGK1), an aldosterone-inducible protein, phosphorylates Nedd4-2 and reduces the binding of Nedd4-2 to EnaC, resulting in increased EnaC expression and activity^65^. E3 Ub-protein ligase WWP1 and E2 Ub-conjugating proteins UBC9, TSG101 also regulate EnaC activity by binding to the α-subunit of EnaC^66^. In summary, if mutations in the α, β, and γ subunits or changes in particular molecules of UPS, UPS may interact with EnaC to change its activity or amount. The increase in the activity or quantity of EnaC induces an increase in sodium reabsorption by epithelial cells, which plays a role in the development of Liddle syndrome. Liddle syndrome has low morbidity and uncommon clinical symptoms, and the diagnosis can only be confirmed by genetic diagnosis, which makes clinical studies of Liddle syndrome more difficult to conduct.

### Renal disease-associated muscle atrophy

One common and serious complication of AKI and chronic kidney disease (CKD) is muscle atrophy. Patients' rates of mortality and morbidity rise linearly as their muscle protein is drastically lost. Research on various metabolic states has demonstrated that an increase in the breakdown of muscle protein by UPS is the cause of the loss of muscle mass in animals or *in vivo* in situations of exuberant catabolism^67^. In organisms undergoing catabolism, the E3 ligases Atrogin-1 (also called MAFbx or FBXO32) and MuRF-1 (also called Trim63) are specifically expressed. MuRF-1 ubiquitinates myosin light chains 1 and 2, myosin heavy chains, myosin-binding protein C, and troponin I, whereas Atrogin-1 interacts with intermediate filament protein vimentin and aligns with the sarcomere Z-disk^68^. Atrogin-1 mRNA levels and muscle proteolysis rate were found to be highly linked in a model of muscle atrophy. Muscle atrophy is accelerated by the activation of the promoters of MuRF-1 and Atrogin-1 by the inflammatory transcription factor NF-κB and the forkhead transcription factor FoxO, respectively. Myostatin, a member of the TGF-β family, inhibits skeletal muscle growth. Myostatin promotes protein degradation by binding to its activating type IIB receptor, which activates the Smad2/Smad3/Smad4 signaling pathway and promotes the expression of Atrogin-1 and MuRF-1^69^. It has been shown that NF-κB promotes myostatin expression, but the exact mechanism remains unclear^70^. In a gentamicin-induced acute kidney injury experiment in rats, expression of MuRF1 and MAFbx was increased in muscle and correlated with AKI severity, whereas the expression of the other three E3 ligases (Nedd4, Trim32, and Fbxo30 / MUSA1) was irrelevant. It is suggested that MuRF1 and MAFbx may be potential targets for the prevention of muscle atrophy during AKI^71^. In complexed or intact myogenic fibers, actin, myosin, troponin, or tropomyosin are all extremely slowly broken down by UPS. The ubiquitination of Muscle protein is accelerated in CKD by caspase-3 and calpain, which break muscle proteins and supply substrates for UPS. In conclusion, E3 ligases may accelerate the degradation of muscle and exacerbate muscular atrophy in cases of AKI or CKD. However, the precise signaling pathways by which UPS controls muscle atrophy in AKI and CKD, require more explanation.

## Proteasome inhibitors in renal disease

Given the significant role that UPS plays in the development of renal disorders, many renal therapies targeting UPS have also been found to have good potential for application, such as proteasome inhibitors, represented by bortezomib. There is a growing body of research on proteasome inhibitors (**Table 2**). In renal diseases, proteasome inhibitors may delay renal injury by inhibiting cell proliferation and differentiation and reducing the expression of NF-κB-dependent inflammatory factors^1^.

### Lupus nephritis (LN)

Zetomipzomib (KZR-616) is a selective immunoproteasome inhibitor that has been the subject of numerous clinical studies in autoimmune diseases. In a mouse model of systemic lupus erythematosus nephritis, KZR-616 persistently reduced proteinuria and renal IgG deposition by regulating signaling processes in lymphocytes and modulating both innate and adaptive immune responses. Zetomipzomib may slow the progression of lupus nephritis by blocking the production of inflammatory factors, but its exact mechanism needs further study^72^. In a clinical study, bortezomib was found to minimize proteinuria and improve renal symptoms in patients treated with lupus nephritis over an extended period. Patients with severe systemic lupus erythematosus who are ineffective on immunosuppressants can combine bortezomib and steroids, which are well tolerated and effective in long-term use^73^. However, a significant proportion of patients treated with bortezomib developed side effects such as infections, and approaches to reduce bortezomib-induced hypogammaglobinemia and lessen adverse effects should be considered in future studies.

### Kidney transplantation

Bortezomib, carfilzomib, and ixazomib all reduce the adverse effects of renal transplantation. Combination treatment with bortezomib and co-stimulation blockade preserved renal function, minimized antibody-mediated rejection (AMR), and extended graft kidney survival after kidney transplantation in a sensitized nonhuman monkey kidney transplant model^74^. In a highly sensitized rhesus monkey kidney transplantation model, the combination of carfilzomib and belazepam was well tolerated and had few side effects. This regimen normalized grafted kidney function and prolonged grafted kidney survival by two months by effectively treating AMR and reducing serum donor-specific antibody (DSA)^75^. In a prospective clinical study, a therapy using carfilzomib in combination with plasma exchange depleted plasma cells and reduced HLA antibodies in highly sensitized renal transplant recipients. It suggests that carfilzomib may reduce AMR responses, but further studies are needed to investigate the mechanisms and to validate its safety and efficacy^76^. In a phase II clinical study enrolling 10 patients awaiting renal transplantation, the combination of ixazomib with other immunosuppressive agents prior to renal transplantation may be effective for desensitization therapy in renal transplantation, but there is heterogeneity in different patients^77^. Because of the differences between animal models of AMR after renal transplantation and patients, more clinical trials are still needed in the future to validate the safety and efficacy of proteasome inhibitors alone or in combination with co-stimulatory blocking agents for AMR.

### Hereditary leiomyomatosis and renal cell carcinoma (HLRCC)

Hereditary leiomyomatosis and renal cell carcinoma (HLRCC) is an autosomal dominant disorder characterized by mutations in the FH gene that result in altered activity of fumarate hydratase, which is involved in the tricarboxylic acid cycle. Patients with HLRCC are at risk of developing aggressive renal cell carcinoma and have a poor prognosis. Marizomib may have a role in HLRCC. In the HLRCC-derived FH-deficient cell line UOK262, marizomib interfered with the metabolism of HLRCC cells. By reducing the expression of lactate dehydrogenase A in tumor cells via p62 and c-Myc, marizomib suppresses glycolysis. Marizomib also modifies glutamine metabolism by reducing the expression of the glutaminases GLS and GLS2, which in turn affects the antioxidant reduction ability of tumor cells^78^. As this trial focused on the effects of marizomib in kidney cancer cells, future studies may also explore the effects of Marizomib on other tumor cells associated with HLRCC. The effect of marizomib on the metabolism of tumor cells may be considered when developing treatment regimens for HLRCC.

### Diabetic kidney disease (DKD)

In a mouse model of DKD, MG-132 inhibited the release of inflammatory molecules and the proliferation of tethered cells, attenuated urine protein, and delayed the degradation of renal function by inhibiting the activation of Akt (serine/threonine kinase) phosphorylation^79^. In diabetic kidney disease, MG132 may also protect renal function by inhibiting the proteasomal degradation of Nrf2 and activating antioxidant genes. MG132 may also attenuate renal inflammatory responses by increasing the stability of IκB, inhibiting the entry of NF-κB into the nucleus, and suppressing the NF-κB signaling pathway^80^. By suppressing IκBα activity, the proteasome inhibitor PSI may also prevent the development of diabetic kidney disease^81^.

A few Chinese herbal remedies might be beneficial for diabetic kidney disease as well. Pioglitazone, quercetin, and Danshen Dripping Pill can all help to improve glucose-lipid metabolism, lower oxidative stress, and prevent the activation of the NF-κB pathway through UPS, which in turn lowers urine protein, reduces renal inflammatory response, and slows down the course of diabetic kidney disease^82^. However, further clinical studies are needed to verify their efficacy.

### Renal fibrosis

Delanzomib specifically caused myofibroblast death in a mouse model of unilateral ureteral obstruction (UUO). This led to a reduction in the expression of the fibrosis genes actin-alpha-2 (ACTA2) and collagen-type-1-alpha-1 (COL1A1) in the kidneys, suggesting that the inhibitor may have therapeutic promise for treating renal fibrosis^83^. Future research may look into Delanzomib's function in other renal fibrosis models, such as IRI and adriamycin.

By targeting UPS, proteolysis-targeted chimeras (PROTACs) also have kidney-protective effects in addition to proteasome inhibitors. PROTACs, which facilitate the breakdown of target proteins by UPS, are made up of an E3 ligase and a target protein joined by a linker. Excellent therapeutic effects of PROTACs have been observed in renal disorders, including AKI, renal cell carcinoma (RCC), and renal fibrosis. In the IRI model, PROTAC NP8 protects renal function by targeting histone deacetylase 6 (HDAC6), increasing the level of the renal protective protein klotho, and decreasing oxidative damage and apoptosis of renal tubular epithelial cells^84^. In RCC, PROTAC PpD promotes the ubiquitinated degradation of P21-activated kinase-4 (PAK4), which in turn inhibits tumor proliferation and increases tumor cell death by immune cells^85^. In renal fibrosis, the TGF-β1/Smad3 signaling pathway is inhibited by PROTAC, which creates a PROTACS that links the E3 ligases VHL and Smad3. The ubiquitination process of the renoprotective factor HIF-2 α is prevented and the level of HIF-2 α expression is enhanced as a result of the interaction between VHL and PROTACS. The nephroprotective effect of the PROTAC has been confirmed in major nephrectomy and UUO models^86^. In addition, leucine-rich α-2 glycoprotein 1 (LRG1) is elevated in renal fibrosis and promotes renal fibrosis through the TGF-β1/Smad3 signaling pathway. PROTAC ^ET^TAC-2 plays a significant role in nephroprotection through the promotion of the degradation of LRG1 and inhibits the process of renal fibrosis^87^.

Promising outcomes are being found in the therapy of many kidney disorders by proteinase inhibitors and PROTACs that target UPS; however, the majority of these studies are currently being carried out in animal models. In order to confirm their safety and effectiveness, more clinical trials are required.

## Ubiquitinase and deubiquitinases

Ubiquitination is a reversible process. Deubiquitinating enzymes (DUBs, also known as deubiquitylases or deubiquitinases) can reverse the ubiquitination process by identifying and removing ubiquitinated proteins from the ubiquitinated substrate proteins and are involved in ubiquitin recycling, editing, and rearrangement^88^. Recent research has shown that the UPS plays a crucial role in kidney diseases. DUBs remove ubiquitin from precursor proteins by either interacting with a large area surrounding the hydrophobic region of ubiquitin or by selectively identifying and hydrolyzing ester, peptide, or isopeptide linkages at the carboxy-terminal end of ubiquitin. DUBs also inhibit E3 ligases by hydrolyzing lysine residues on target proteins with the peptide bond at the C-terminal end of ubiquitin^89^. By working on distinct substrate proteins, DUBs play a role in several biological processes, including cell proliferation, differentiation, and apoptosis. DUBs can be classified into six families: ubiquitin-specific proteases (USPs), ovarian tumor-associated proteases (OTUs), ubiquitin carboxy-terminal hydrolases (UCHs), Machado-Josephin domain proteases (MJDs), Mindys family motif interacting with ubiquitin containing novel dub family (MINDYs), and JAB1 / MPN / MOV34 metalloproteinases (JAMMs)^90^. The balance between ubiquitination and deubiquitination is finely regulated to maintain protein homeostasis and normal organismal function. The imbalance between ubiquitination and deubiquitination may be associated with the pathogenesis of a variety of diseases, including tumors^91^, hematologic disorders^92^, and neurodegenerative diseases^93^. DUBs are involved in several disorders of the kidneys.

### Deubiquitinases in diabetic kidney disease (DKD)

The deubiquitinating enzymes UCH-L1, USP22, OTUD5, and USP14 act on PIRK3, TAK1, and SPAG5, respectively, and play a regulatory role in the pathophysiological process of diabetic kidney disease (**Table 3**). Renal biopsy samples from patients with DKD and high glucose-induced podocytes have increased expression of UCH-L1, which may be mediated by the Wnt/β-catenin signaling pathway. This results in decreased expression of the articulin CD2AP and the podocyte cytoskeleton protein synaptopodin, while increased expression of the transcription factor snail and nerphrin, a marker protein of podocyte injury, ultimately leads to podocyte actin skeleton remodeling and cell migration^94^. Elevated glucose levels trigger the overexpression of UCHL1 in podocytes, which in turn promotes the deubiquitination of RIPK1 and RIPK3, which results in necroptosis of podocytes, causing podocyte depletion^95^. Therefore, targeting UCH-L1 to inhibit the RIPK1/RIPK3 signaling pathway may be a viable option to delay necrotic apoptosis under high glucose conditions. In diabetic rat kidneys and high-glucose cultured podocytes, USP22 was increased. USP22 contributes to the inflammatory response and apoptosis in podocytes through oxidative stress and secretion of inflammatory mediators, up-regulation of caspase-3, and an increase in the BAX/Bcl-2 ratio^96^. USP22 also deubiquitinates RIPK3, which causes high hyperglycemia to trigger damage to podocyte intercellular collagenization while targeting USP22 increased the amount of ubiquitinated degradation of RIPK3 and protected podocytes^97^. This suggests that USP22 may play a role in podocyte injury through RIPK3, but the exact mechanism needs to be further investigated. USP22 may also be involved in tubulointerstitial fibrosis in DKD by affecting the expression of Snail. Snail is an important transcription factor that affects EMT, and Snail promotes EMT by repressing E-calmodulin expression and up-regulating Vimentin and FN expression. USP22 promotes EMT by stabilizing Snail expression, further affecting tubulointerstitial fibrosis^98^. Further studies found that quercetin can inhibit the USP22/Snail signaling pathway and delay the progression of DKD^99^. The expression of OTUD5 was decreased in podocytes under high glucose stimulation. The overexpression of OTUD5 protects podocytes. OTUD5 inhibits the inflammatory response of podocytes by deubiquitinating TAK1 and inhibiting the phosphorylation activation of TAK1, which reduces podocyte damage and delays DKD^100^. Thus, enabling podocytes to overexpress OTUD5 may be a potential therapeutic strategy to delay podocyte inflammation through the OTUD5-TAK1 axis. Research has demonstrated that USP14 can regulate the deubiquitination of SPAG5 and stimulate the up-regulation of SPAG5 expression in podocytes. This, in turn, activates AKT/mTOR signaling to inhibit autophagy, causing injury to podocytes^101^. In conclusion, UCH-L1, USP22, OTUD5, and USP14 are promising therapeutic targets in DKD, but more clinical studies are needed to confirm safety and efficacy.

### Deubiquitinases in clear cell renal cell carcinoma (ccRCC)

USP13, USP53, and OTUD1 are involved in the growth and metastasis of clear cell renal cell carcinoma (ccRCC). It was found that USP13 could deubiquitinate zinc fingers and homeoboxes 2 (ZHX2) and stabilize ZHX2 protein. Specific USP13 knockdown in tumor cells can inhibit tumor growth, suggesting that USP13 inhibitors may have therapeutic effects on ccRCC^102^. Conversely, USP53 knockdown encourages the development of tumor cells. By deubiquitinating IκBα, USP53 prevents NF-κB activation, suppresses the NF-κB signaling pathway, and inhibits ccRCC^103^. Additionally, the degree of malignancy of renal cancer cells is correlated with the expression level of the USP53 gene, indicating that USP53 could be a viable target for gene therapy of renal carcinoma. Reduced expression of OTUD1 in renal cancer patients raises PTEN ubiquitination degradation, which stimulates the TNF-α/NF-κB and PI3K/AKT signaling pathways, encourages the growth of ccRCC, and influences patient prognosis. TKIs are the first-line therapeutic agents for ccRCC, however, most patients are susceptible to resistance after treatment with TKIs. Studies have shown that OTUD/PTEN can affect the resistance of ccRCC patients to TKIs, and that inhibition of OTUD1 can increase the sensitivity of ccRCC to TKIs. Although the specific mechanisms affecting OTUD1 expression remain to be further investigated, existing studies suggest that OTUD1 may be an important factor influencing the prognosis and drug resistance of ccRCC patients^104^.

### Deubiquitinases in other renal diseases

In AKI, glycolysis affects macrophage M1-like polarization and its immune function. Increased expression of USP25 in AKI affects PKM2 (M2 isoform of pyruvate kinase, muscle), a crucial enzyme for glycolysis, which in turn causes kidney damage in AKI^105^. In contrast, in a hypertensive nephropathy model, USP25 inhibited the TGF-β signaling pathway by suppressing the ubiquitination process of Smad4, delaying renal fibrosis and protecting renal function^106^.

### Relationship between ubiquitinase and deubiquitinases

In the kidney, ubiquitination and deubiquitination act synergistically to regulate organismal protein homeostasis and influence the progression of renal disease by affecting signaling pathways.

#### Regulates ubiquitination levels

Ubiquitinase and deubiquitinase affect the level of ubiquitination by acting on common substrate proteins. The DUB CYLD inhibits ubiquitinated degradation of IκBα, whereas the E3 ligase Trim29 promotes ubiquitinated modification of IκBα, which affects NF-κB activity and influences DKD progression. Ubiquitination and deubiquitination affect intracellular protein homeostasis by altering the ubiquitin chain of substrate proteins, influencing their degradation by the proteasome, and modulating downstream signaling pathways.

#### Protein interaction

The DUB USP10 interacts with the ubiquitin ligase MDM2 and activates MDM2 by removing the MDM2 polyubiquitination chain. In a mouse model of adriamycin nephropathy, MDM2 deficiency reduces the expression of inflammatory and chemokine factors in the kidney and protects renal function^107^. USP10 also stimulates the deubiquitination of Smurf1 and stabilizes it. Smurf1 has a key function in both DKD and renal fibrosis. Additionally, by reducing the expression of Smad1 and Smad5, Smurf1 inhibits the TGF-β/BMP signaling pathway, promotes cell proliferation, and is crucial for the development of renal tissue^108^. In conclusion, ubiquitinases and deubiquitinases can influence protein homeostasis and regulate the development of renal disease by acting on common substrate proteins or by interacting with each other.

## Ubiquitinase and autophagy

**Figure 3:**
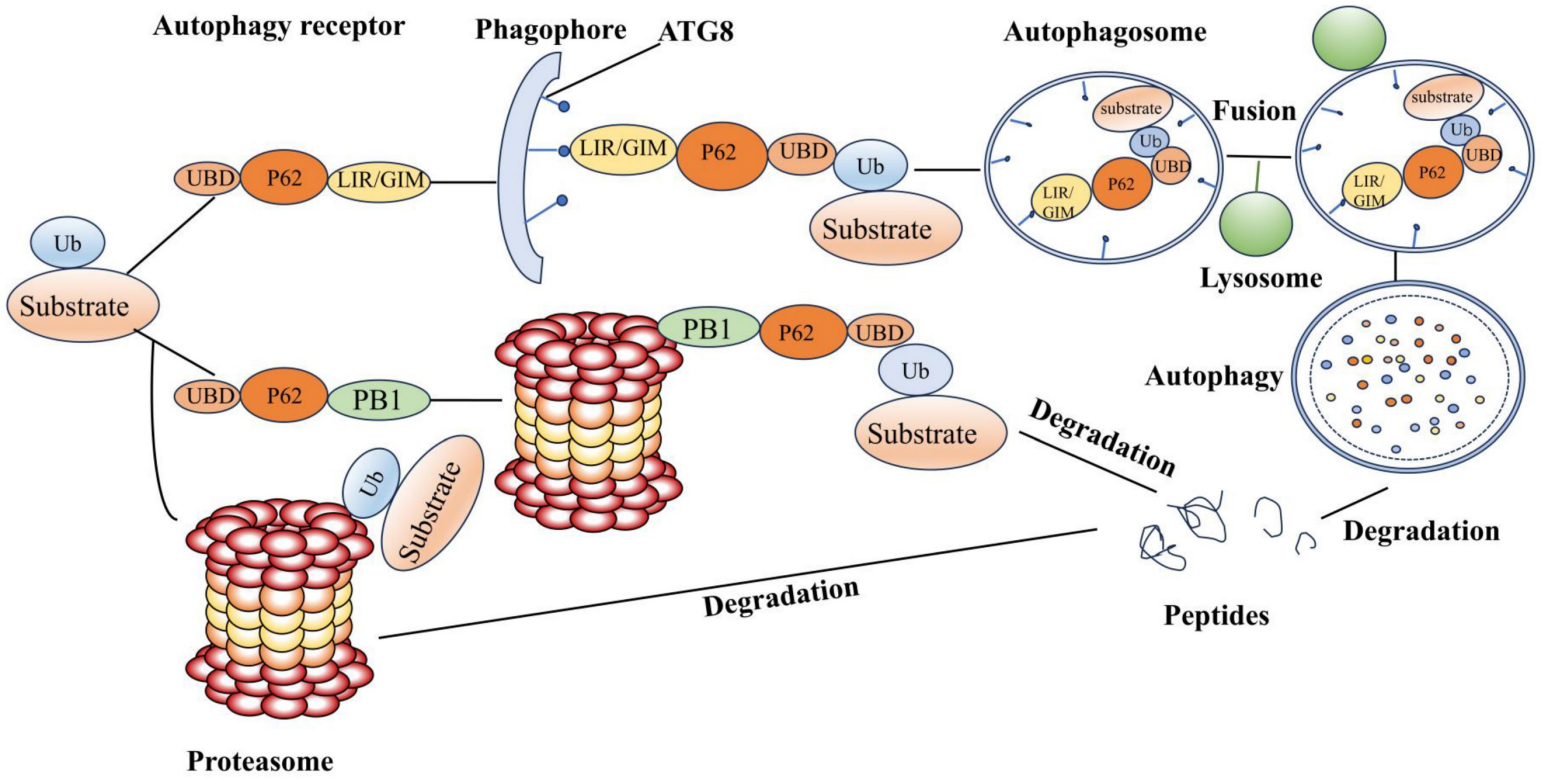
** The connection between autophagy and UPS.** P62/SQSTM1, the most studied autophagy receptor, binds to autophagic membranes through its LIR or GIM structural domains, to the proteasome through its Phox and Bem1p structural domains (PB1), and to ubiquitinated substrates through its Ub-associated structural domain (UBA). The ubiquitinated substrate can be identified by the UBA structure on P63 that contains the PB1 structural domain, or directly by the proteasomal intrinsic ubiquitin receptor. Following recognition, the substrate is deubiquitinated, unfolded, and eventually broken down by the proteasome. Via UBA, P63 containing the LIR or GIM structure domain attaches to ubiquitinated protein aggregates, mitochondria, etc., and the LIR or GIM structural domain binds to LC3 or GABARAP on the autophagy membrane and then forms autophagosomes. Autophagy, which facilitates substrate breakdown, is created when autophagosomes attach to lysosomes. P62 is therefore a crucial receptor for controlling autophagy and UPS.

UPS and autophagy are two major protein degradation pathways found in eukaryotic cells. Autophagy is the intracellular transportation of substances to be degraded to the lysosome for degradation, and it is divided into three types according to physiological functions and transportation pathways, macroautophagy, microautophagy, and molecular chaperone-mediated autophagy. The main target of autophagy is the degradation of large, long-lived proteins. Energy shortage and hunger are the primary inducers of autophagy, which aims to restore the intracellular supply of nutrients like ATP and amino acids for protein synthesis. The degree of cellular autophagy depends primarily on the kind of cell and the availability of nutrients. The activation of ULK1 kinase complexes, which facilitates the binding of the autophagy-associated protein 8 ATG8 protein family (also known as LC3s and GABARAPs) to phosphatidylethanolamines or phosphatidylserines and anchors them to autophagic membranes, is what initiates autophagy^109^. The GABARAP interacting motif (GIM) or the LC3 interacting region (LIR) accepts substrates delivered by autophagy receptors. UPS and autophagy regulation are significantly influenced by the ubiquitin and autophagy receptor p62/SQSTM1 (**Figure 3**). After the substrate is ubiquitinated, competitive binding of the ubiquitin receptor to the proteasome and ATG8 determines whether the substrate is degraded via UPS or ALS. The affinity of the ubiquitin receptor for the proteasome pathway is stronger, but as the substrate aggregates, the autophagy receptor increases its affinity for ubiquitin. Inhibition of UPS induces autophagy, which degrades ubiquitinated and misfolded proteins. The two systems work together to preserve cell homeostasis by destroying unnecessary and damaged proteins and regulating the cell cycle, DNA transcription, and translation.

### Autophagy in kidney

Cellular autophagy not only plays an important role in kidney development and maintenance of homeostasis of podocytes, endothelial cells, and tubular epithelial cells but also has been implicated in the pathogenesis of several renal diseases. Autophagy was shown to mitigate AKI in a mouse model of IRI indicating that autophagy protects proximal tubular epithelial cells and that tubular epithelial cell injury was worsened by targeted suppression of the Atg5 gene^110^. High hyperglycemia causes RTECs to over-activate mTOR and Smad3, inhibit activation of TEFB and transcription, and prevent lysosome neogenesis, which ultimately leads to lysosome exhaustion and the incapacity of autophagosome breakdown by lysosomal enzymes^111^.

Mitophagy can also play a role in several acute kidney injury models. In I/R-AKI, N-myc downstream-regulated gene 2 (Ndrg2) deficiency can reduce oxidative stress and delay renal injury by activating PINK1/1Parkin-mediated mitophagy, but the exact mechanism remains to be investigated further^112^. In LPS and CLP-induced AKI models, renal tubular cells can protect renal function by inducing PINK1/Parkin-mediated mitochondrial autophagy, suggesting that the PINK1/Parkin pathway may be an effective therapeutic target for AKI^113^. In contrast-induced acute kidney injury (CI-AKI), the PINK1/Parkin pathway also protects RTECs from apoptosis by reducing oxidative stress and inhibiting nucleotide-binding oligomerization domain-like pyrin domain containing protein 3 (NLRP3) inflammasome production^114^.

### Relationship between ubiquitination and autophagy

UPS and autophagy interact in the pathogenesis of DKD, focal segmental glomerulosclerosis (FSGS), and ccRCC, and jointly regulate disease progression.

#### Diabetic kidney disease (DKD)

STX17 and SNAP29 are autophagosomal membrane proteins that can be degraded by the 20s proteasome in a nonubiquitinated manner. Enhanced proteasome activity inhibits autophagy by increasing the degradation of STX17 and SNAP29 and preventing the fusion of autophagosomes and lysosomes^115^. This effect may have led to a compensatory upregulation of autophagy upon inhibition of UPS, explaining why proteasome inhibitors are used to delay the course of DKD.

#### Focal segmental glomerulosclerosis (FSGS)

Studies have shown that African populations are more susceptible to FSGS due to the presence of variants of apolipoprotein L1 (APOL1). APOL-1 alleles APOL-G1 and APOL-G2 specifically damage podocytes and promote kidney injury^116^. Autophagy is protective against FSGS. APOL1 protein promotes kidney injury by depolarizing the autophagic membrane to inhibit the fusion of autophagosomes and lysosomes^117^. The UBD gene can produce a ubiquitin-like protein that modifies APOL-G1 and APOL-G2 proteins to promote their lysosome breakdown and reduce their cytotoxicity^118^.

#### Clear cell renal cell carcinoma (ccRCC)

HIF-2α is an oncogenic transcription factor in ccRCC, and its accumulation stimulates the development of tumors. Research has indicated that HIF-2α is degraded by both ALS and UPS and that ALS inhibition increases UPS degradation whereas UPS inhibition increases UPS degradation of HIF-2α^119^. Modulation of the activities of UPS and ALS may contribute to the treatment of ccRCC. In conclusion, common substrates, (de)ubiquitinating enzymes, common regulatory kinases, ubiquitin receptors, transcription factors, and pathways are some of how the two systems are connected^120^. Thus, when developing treatment plans for renal disorders, consideration should be given to the UPS-ALS interaction mechanism.

## Conclusion and prospects

UPS plays an important role in the pathophysiological process of renal diseases by regulating the activity of downstream signaling pathways and participating in oxidative stress, inflammation, apoptosis, and other injury processes. Deubiquitination works with UPS to maintain protein homeostasis in organisms by affecting the level of ubiquitination of substrates and influencing the degradation of proteins by UPS. Autophagy and UPS are mainly connected through a common ubiquitinated substrate. In the physiological state, UPS is the main protein degradation pathway. When cellular injury and oxidative stress occur, UPS and autophagy can undergo compensatory effects to regulate cellular homeostasis. UPS interacts with deubiquitination and cellular autophagy, constituting a complex signaling regulatory network, which together regulate the development of renal diseases.

Although there are more and more animal experiments on UPS modulating renal diseases, further exploration of its specific mechanism is needed. Since the majority of present research on UPS is preclinical, more work must be done to confirm its precise mechanism in human illnesses. It has been discovered that proteasome inhibitors and co-blocking medications may be helpful in treating renal disease; however, more research is required to determine the safest and most efficient way to combine these two classes of drugs to maximize disease improvement and reduce side effects. The majority of the present research has been done on the entire kidney; however, to reduce the negative effects of medications caused by interactions between different renal cells, future studies may assess the function of the UPS in certain renal cells. UPS can interact with other systems and certain E3 ligases can play a role in a variety of diseases. For example, Smurf1 plays a role in various diseases such as DKD and renal fibrosis and can interact with deubiquitinating enzymes to influence disease progression. Therefore, future research could be focused on finding UPS-specific targets within specific cells. By identifying UPS-specific markers concerning kidney disease, drugs targeting particular components of the UPS rather than the entire UPS could be developed to minimize adverse drug reactions.

## Figures and Tables

**Table 1:** E3 Ub-protein ligase in diabetic kidney disease

E3 Ub-protein ligase	Substrate	Model	Target cell	Reference
VHL	Glucose-6-phosphate dehydrogenase	Renal biopsy samples in diabetic kidney disease and STZ-induced diabetic mouse models	Podocyte	33
Trim63	PPARα	Db/db mouse models and ARD, high glucose, mannitol, Wnt3a-treated mouse podocyte	Podocyte	34
NEDD4L	IKK	STZ-induced diabetic mouse models and low glucose, high glucose, mannitol-treated human podocyte	Podocyte	35
Trim29	IκBα	High glucose-treated mouse podocyte	Podocyte	36
APC/C	Cyclin B1, Skp2	Renal biopsy samples in diabetic kidney disease and db/db mouse models	Podocyte	37
Parkin	GATA4	Renal biopsy samples in diabetic kidney disease and STZ-induced mouse models	Renal tubular epithelial cell	40
FBXW7	SreBP-1	STZ-induced mouse models	Renal tubular epithelial cell	41
Trim13	CHOP	Renal biopsy samples in diabetic kidney disease, STZ-induced mouse models, db/db mouse models, and high glucose-treated mouse glomerular mesangial cells	Glomerular mesangial cell	45
Smurf1	TGR5	STZ-induced mouse models, db/db mouse models	Glomerular mesangial cell	46

**Table 2:** Proteasomal inhibitors in renal diseases

Proteasome inhibitors	Disease	Model	Function	Reference
Zetomipzomib (KZR-616)	Lupus nephritis	Lupus nephritis mouse model	Reduce proteinuria and anti-dsDNA antibodies	72
Bortezomib	Lupus nephritis	2014-2020 SLE patients treated with Bortezomib at Swedish University Hospitals	Reduce proteinuria and seroconversion of antibodies	73
Bortezomib	Kidney transplantation	Non-human primate kidney transplantation model	Reduce antibody-mediated rejection	74
Carfilzomib (PR-171)	Kidney transplantation	Nonhuman primate kidney transplantation model	Reduce serum donor-specific antibody levels and improve kidney graft function	75
Carfilzomib (PR-171)	Kidney transplantation	Thirteen highly sensitized kidney transplant candidates at the Christ Hospital and the University of Cincinnati, in Cincinnati, OH	Deplete of plasma cells and reduce HLA antibodies	76
Ixazomib	Kidney transplantation	Phase II clinical trial of highly sensitized renal transplant candidates	May induce renal graft desensitization	77
Marizomib	Hereditary leiomyomatosis and renal cell carcinoma	UOK262-induced renal tumor mice model	Inhibit the metabolism of glutamine and glucose	78
MG132	Diabetic kidney disease	STZ-induced diabetic mouse models	Inhibit renal inflammation, mesangial cell proliferation, and renal fibrosis	79
MG132	Diabetic kidney disease	STZ-induced diabetic mouse models	Inhibit oxidative damage and inflammatory responses in the kidney	80
PSI	Diabetic kidney disease	STZ-induced diabetic mouse models	Inhibit NF-κB signaling pathways	81
Delanzomib	Renal fibrosis	Unilateral ureteric obstruction mouse model	Induce myofibroblast death	83

**Table 3:** Deubiquitinating enzymes in diabetic kidney disease

Deubiquitinating enzymes	Substrate	Model	Reference
USP22	Sirt1	Glomerular mesangial cells cultured in high-sugar	42
USP25	TRAF6	STZ-induced diabetic mouse models	43
CYLD	IκBα	High glucose-treated glomerular mesangial cells	44
UCH-L1	RIPK1, RIPK3	Renal biopsy samples in diabetic kidney disease and high glucose-treated podocytes	95
USP22	RIPK3	High-sugar cultured podocytes	97
OTUD5	TAK1	STZ-induced diabetic mouse models	100
USP14	SPAG5	High glucose-treated podocytes	101

## Abbreviations

ACOX1: acyl-coenzyme A oxidase 1
ACTA2: actin-alpha-2
ADR: adriamycin
AGEs: advanced glycation-end products
AKI: acute kidney injury
ALS: autophagosomal lysosomal system
AMR: antibody-mediated rejection
BAP1: breast cancer susceptibility protein 1 (BRCA1)-associated protein 1
ccRCC: clear cell renal cell carcinoma
CHOP: C/EBP homologous protein
CHK1: checkpoint kinase 1
CKD: chronic kidney disease
CLP: cecal ligation and puncture
COL1A1: collagen-type-1-alpha-1
CP: core particle
CPT1A: carnitine palmitoyl transferase 1a
DKD: diabetic kidney disease
DNMT1: DNA methyltransferase
DSA: donor-specific antibody
DUBs: deubiquitinating enzymes
Dvl2: Dishevelled-2
ECM: extracellular matrix
EGFR: epidermal growth factor receptor
EMT: epithelial-mesenchymal transition
ET-1: endothelin 1
FAO: fatty acid oxidation
FBXW7: F-box and WD repeat domain-containing 7
FMR1: FMRP translational regulator 1
FN: fibronectin
FSGS: focal segmental glomerulosclerosis
FSP1: ferroptosis suppressor protein 1
GIM: GABARAP interacting motif
GMCs: glomerular mesangial cells
GPX4: glutathione peroxidase 4
G6PD: glucose-6-phosphate dehydrogenase
HDAC6: histone deacetylase 6
HLRCC: hereditary leiomyomatosis and renal cell carcinoma
HRD1: HMG-CoA reductase degradation protein 1
IKK: IκB kinase complex
IRI: ischemia-reperfusion injury
LIR: LC3 interacting region
LPS: lipopolysaccharide
LRG1: leucine-rich α-2 glycoprotein 1
LRP-5/6: lipoprotein receptor-related protein-5/6
PAK4: P21-activated kinase-4
PINK1: PTEN-induced kinase1
PPARα: peroxisome proliferator-activated receptor α
PROTACs: proteolysis-targeted chimeras
RCC: renal cell carcinoma
RP: regulatory particle
RTECs: renal tubular epithelial cells
SCF: Skp1-Cullin1-F-box
Sirt1: silent information regulator 2-related protein 1
SreBP-1: sterol regulatory element-binding protein 1
TBK1: TANK-binding kinase 1
TLR4: toll-like receptor 4
TNFAIP8L1/TIPE1: tumor necrosis factor alpha-induced protein 8-like 1
Ub: ubiquitin
UPS: ubiquitin-proteasome system
UUO: unilateral ureteral obstruction
ZHX2: zinc fingers and homeoboxes 2
